# *The Organon* and Materia Medica Club of the Bay Cities of California

**Published:** 1896-06

**Authors:** W. E. Ledyard

**Affiliations:** Secretary


W. E. LEDYARD, *Secretary.*

Reported by Eleanor F. Martin, M. D.

---

### *THE ORGANON* AND MATERIA MEDICA CLUB OF THE BAY CITIES OF CALIFORNIA.

The regular semi-monthly meeting was held at the office of Dr. George H. Martin, The Wenban, 606 Sutter Street, Friday evening, February 7th, 1896.

Members present: Drs. J. M. Selfridge, W. E. Ledyard, A. McNeil, M. T. Wilson, G. J. Augur, and M. F. Underwood.

The meeting was called to order at 8.30 by the President, Dr. J. M. Selfridge. The minutes of the previous meeting were read by the Secretary, and after corrections by Drs. Selfridge, were approved. Most of the evening was taken up by a discussion on expert testimony by Dr. Selfridge concerning a case on trial in Oakland, after which Dr. McNeil was appointed reader of the evening, who commenced *The Organon* at Section 60–61.

### DISCUSSION.

Dr. McNeil—Did I understand in the minutes of the previous meeting just read that Dr. Martin stated that Morphine and the homœopathic remedy could be given at the same time?

Dr. Selfridge—No. What Dr. Martin meant was that if Morphine had been given, the homœopathic remedy would also act.

Dr. McNeil—That would be claiming that the homœopathic remedy is more powerful than the Morphine, and I have not found it so. If the case is taken before the palliative is given, we do not allow for the symptoms produced by the palliative, and *vice versa*.

Dr. Selfridge—Here is a case for illustration: A man with severe hemorrhage of the nose. Liquid Monsell's Salts was used liberally. The nose was plugged from the throat to the nostril, and yet he would bleed profusely. The galvanic cautery was used, and still he bled. The patient was given Ergot until he was ergotized, and still he bled. We thought he would die. Dr. Clarence Selfridge gave one dose of *Aconite*$^{200}$, and the hemorrhage stopped. The great restlessness, dry, hot skin, bounding full pulse, and fear of death of Aconite were present. The dose was repeated occasionally. Homœopathic remedies will act in spite of other drugs.

Dr. Underwood—If this were not so, how could we account for the wonderful cures of allopathized patients?

Dr. McNeil—This should not be used, however, as an excuse for giving palliatives. In Dr. Selfridge's case there still remained a picture of Aconite, which is not always so when a palliative is given, as the mere crude drug covers up the symptoms of the indicated homœopathic remedy.

Dr. Selfridge—I do not approve of using hypodermics of Morphine before giving the homœopathic remedy, but I know that the remedy will act even if the Morphine has been used, providing the proper remedy is given.

Dr. Augur—As in taking *Natrum-mur*. when we use plenty of salt every day.

Dr. Wilson—Notwithstanding the allopathic drugging the indicated remedy will act.

Dr. McNeil—I know that we do make cures in spite of these things, but all the more reason why we should try to prevent the use of palliatives. There is a possibility that accidental cures have been made when patients have been treated allopathically.

Section 62 was read.

The meeting was then declared adjourned, to meet again the third Friday in February, at Dr. Martin's offices.

